# Novel Insights on MRGPRX2-Mediated Hypersensitivity to Neuromuscular Blocking Agents And Fluoroquinolones

**DOI:** 10.3389/fimmu.2021.668962

**Published:** 2021-07-27

**Authors:** Jessy Elst, Marcus Maurer, Vito Sabato, Margaretha A. Faber, Chris H. Bridts, Christel Mertens, Michel Van Houdt, Athina L. Van Gasse, Marie-Line M. van der Poorten, Leander P. De Puysseleyr, Margo M. Hagendorens, Viggo F. Van Tendeloo, Eva Lion, Diana Campillo-Davo, Didier G. Ebo

**Affiliations:** ^1^Department of Immunology, Allergology, Rheumatology and the Infla-Med Centre of Excellence, Faculty of Medicine and Health Sciences, University of Antwerp, Antwerp, Belgium; ^2^Immunology, Allergology, Rheumatology, Antwerp University Hospital, Antwerp, Belgium; ^3^Dermatological Allergology, Allergie-Centrum-Charité, Department of Dermatology and Allergy, Charité - Universitätsmedizin, Berlin, Germany; ^4^Department of Immunology, AZ Jan Palfijn Hospital Gent, Ghent, Belgium; ^5^Department of Pediatrics, Faculty of Medicine and Health Sciences, University of Antwerp and Antwerp University Hospital, Antwerp, Belgium; ^6^Laboratory of Experimental Hematology, Vaccine & Infectious Disease institute (VAXINFECTIO), Faculty of Medicine and Health Sciences, University of Antwerp, Antwerp, Belgium

**Keywords:** MRGPRX2, IgE, mast cell, drug, anaphylaxis, CD63, flow cytometry, rocuronium

## Abstract

Neuromuscular blocking agents (NMBAs) like atracurium and rocuronium as well as fluoroquinolones (FQs) cause mast cell-mediated anaphylaxis by activating Mas-related G protein-coupled receptor X2 (MRGPRX2), but many questions remain unanswered. Here, we address three of them, namely whether primary human mast cells show similar activation by these drugs as murine mast cells and mast cell lines, how sugammadex protects from atracurium-induced MRGPRX2-mediated mast cell activation, and why some but not all patients treated with rocuronium develop anaphylaxis. We used peripheral blood-derived cultured mast cells from healthy donors and patients, assessed mast cell activation and degranulation by quantifying intracellular calcium and CD63 expression, respectively, and made use of MRGPRX2-silencing, *via* electroporation with Dicer-substrate small interfering RNAs, and single cell flow cytometric analyses. Atracurium, ciprofloxacin, and levofloxacin activated and degranulated primary human mast cells, but only MRGPRX2-positive and not MRGPRX2-negative or -silenced mast cells. Sugammadex attenuated the atracurium-induced and MRGPRX2-mediated activation and degranulation of human mast cells by reducing free atracurium levels. The mast cells of patients with IgE-independent anaphylaxis to rocuronium were similar, in their MRGPRX2 expression and function, to those of patients with IgE-mediated anaphylaxis. These findings further improve our understanding of the role and relevance of MRGPRX2-driven mast cell activation in anaphylactic reactions to NMBAs and FQs and may help to improve their prediction, prevention, and treatment.

## Introduction

The activation of Mas-related G protein-coupled receptor X2 (MRGPRX2) on mast cells (MCs) is held to be a major pathway in the pathogenesis of IgE-independent immediate drug hypersensitivity reactions (IDHRs) that occur in response to neuromuscular blocking agents (NMBAs) such as atracurium and rocuronium as well as fluoroquinolones (FQs) such as moxifloxacin and ciprofloxacin. NMBAs and FQs can cause perioperative anaphylaxis, and adverse reactions to these drugs administered during general anesthesia may be very severe and life-threatening, with a mortality rate of up to 9% (1–7).

As of today, many questions on the role of MRGPRX2 in NMBA- and FQ-induced anaphylaxis remain unanswered and need to be addressed. These include, but are not limited to, 1) whether findings on MRGPRX2 activation by NMBAs and FQs from mouse models or genetically modified cell lines can be extrapolated to humans, 2) what the precise role and mechanisms of action of sugammadex, a NMBA reversal agent, are in atracurium-induced and MRGPRX2-induced adverse reactions (8), and 3) why some but not all patients treated with NMBAs experience MRGPRX2-related IDHRs.

At present, most of the information on the effects of NMBAs and FQs on MRGPRX2 are derived from studies performed with MRGPRX2-expressing neoplastic cells, like the LAD-2 MC line, or with the mouse orthologue, MRGPRB2. It is clear that there are differences between these cellular models and primary human MCs. LAD-2 cells, for example, are more responsive to MRGPRX2-agonists than human peripheral blood-derived cultured MCs (PBCMCs) (9, 10), and some NMBAs are less potent activators of MRGPRX2 than MRGPRB2 (11). This makes it difficult, at present, to assess NMBAs and FQs for their MRGPRX2-activating capacity and potency in the human system and to predict the risk that comes with their use. What is needed are results from studies with primary human cells. Such studies, until very recently, were not possible due to the lack of suitable tools, models and assays.

Sugammadex is a modified γ-cyclodextrin designed as a selective relaxant-binding agent that acts by encapsulating rocuronium as an inclusion complex and removing it from the neuromuscular junction (12). Although sugammadex does not reverse atracurium-induced neuromuscular block (13), it has recently been shown to attenuate atracurium-induced MRGPRX2-dependent MC activation in LAD-2 cells, when used at molar excess (14). Currently, the underlying mechanisms of this effect are unclear. The prevention of atracurium-induced MC activation may be caused by atracurium encapsulation, i.e. the elimination of free atracurium, by a direct inhibition *via* sugammadex, or by inhibitory effects of sugammadex-atracurium complexes. Addressing this gap of knowledge may guide the development of more effective approaches for the use of sugammadex for the prevention of NMBA-induced IDHRs.

Although MCs in all humans are held to express MRGPRX2, very few patients develop IDHRs when treated with NMBAs such as rocuronium. The reasons for this are unknown, but may include genetic polymorphisms and mutations resulting in an augmented responsiveness of MRGPRX2, distinct receptor binding sites, differences in MRGPRX2 signalosome, epigenetic modifications, post-transcriptional modifications resulting in synthesis of MRGPRX2 variants, temporarily or constitutively varying surface expressions and the influence of co-factors (15) (10). As recently suggested by Chompunud et al. (10), one way to address the role of MRGPRX2 mutations is to compare MCs for MRGPRX2 expression and function between patients who experienced IDHRs and had a positive skin test rocuronium, with and without rocuronium-specific IgE and/or positive BAT rocuronium (16).

Recently, we and others developed tools, models and assays that allow for the investigation of the three questions at hand. These include a primary human MC model in which silencing of MRGPRX2 *via* DsiRNA electroporation is coupled to flow cytometric analysis (17). In this model, the introduction of *MRGPRX2*-targeting DsiRNA (MRGPRX2-DsiRNA) almost completely mitigates intracellular calcium elevations and/or degranulation in response to MRGPRX2 ligands such as the opiate morphine, the FQ moxifloxacin, and the NMBA rocuronium (17). Here, we made use of these novel instruments to address important and unanswered questions on the role of MRPRX2 in NMBA- and FQ-induced IDHRs.

## Materials and Methods

### Generation of Human Peripheral Blood-Derived Cultured Mast Cells (PBCMCs)

Human PBCMCs were generated as described previously (18). Briefly, peripheral blood mononuclear cells (PBMCs) were isolated from blood samples of healthy donors or rocuronium-hypersensitive patients using Histopaque. Next, CD34^+^ progenitors were isolated from PBMCs (EasySep Human CD34 Selection Kit, Stemcell Technologies) and cultured, for 4-5 weeks, in serum-free methylcellulose-based medium (MethoCult SF H4236, Stemcell Technologies) supplemented with penicillin (100 units/mL, Life Technologies), streptomycin (100 μg/mL, Life Technologies), low-density lipoprotein (LDL, 10 μg/mL, Stemcell Technologies), 2-mercaptoethanol (55 μmol/L, Life Technologies), stem cell factor (SCF, 100 ng/mL, Miltenyi Biotec), interleukin-3 (IL-3, 100 ng/mL, PeproTech) and interleukin-6 (IL-6, 50 ng/mL, Miltenyi Biotec). PBCMCs harbor a MRGPRX2^+^ and a MRGPRX2^-^ subpopulation (**Supplementary Figure 1**). Each PBCMC culture yielded 75 ± 5% MRGPRX2^+^ cells (n=15).

### Assessment of Mast Cell Activation by Intracellular Calcium Staining

For intracellular calcium staining, PBCMCs at a concentration of 5x10^5^ cells/mL, were loaded with 1 µM Fluo-4 AM (ThermoFisher Scientific) for 45 min at 37°C, washed with phosphate-buffer saline (PBS, ThermoFisher Scientific), and resuspended in 300 µL pre-warmed (37°C) Tyrode’s buffer (Sigma-Aldrich). The intensity of Fluo-4 AM was measured at a single cell level for 50 seconds without stimulation. Next, the cells were stimulated with Tyrode’s buffer as negative control, substance P (74 µM, Sigma Aldrich) as positive control for the MRGPRX2 pathway, anti-FcϵRI as a positive control for the IgE-pathway (2.5 µg/mL, ThermoFisher Scientific), succinylcholine (Celocurine^®^, CSP Benelux), atracurium (Tracrium^®^; Aspen Pharma), ciprofloxacin (Bayer) or levofloxacin (Fresenius Kabi), followed by immediate further reading for 2 min. Dose-response experiments (**Supplementary Figure 2**) were done with succinylcholine (27.7 µM, 277 µM, 1384 µM, 2768 µM and 5536 µM), atracurium (10.8 µM, 108 µM, 538 µM, 1076 µM and 2152 µM), ciprofloxacin (15.1 µM, 151 µM, 755 µM and 1510 µM) and levofloxacin (13.8 µM, 138 µM, 692 µM, 1384 µM and 2767 µM). Optimal stimulation concentrations were 5536 µM for succinylcholine, 2152 µM for atracurium, 755 µM for ciprofloxacin (1510 µM appeared to be cytotoxic) and 2767 µM for levofloxacin. Combining the staining of MRGPRX2 and intracellular calcium comprised first staining of the membrane expression of MRGPRX2 for 20 min at 4°C, after which the cells were washed with PBS and stained with Fluo-4 AM according to the protocol described above.

### Assessment of Mast Cell Degranulation by CD63 Up-Regulation

For CD63 measurements, PBCMCs, defined as CD117^+^ and CD203c^+^ cells, were dissolved in pre-warmed (37°C) Tyrode’s buffer at a concentration of 5x10^5^ cells/mL. Next, 100 μL of the cells were stimulated with 100 µL tyrode buffer, anti-FcϵRI, substance P, succinylcholine, atracurium, ciprofloxacine or levofloxacin, for 3 and 20 min at 37°C. Reactions were stopped by placing the cells on ice. Subsequently, supernatants were removed by centrifugation (500 x g, 4°C, 5 min). Cells were stained with anti-human CD117-APC (clone 104D2, BD Biosciences), anti-human CD203c-PECy7 (clone NP4D6, eBioscience), anti-human MRGPRX2-PE (clone K125H4, BioLegend) and anti-human CD63-FITC (clone H5C6, BD Biosciences) for 20 min at 4°C. Next, cells were fixed with 1 mL Phosflow Lyse/Fix buffer (BD Biosciences) for 20 min. Finally, cells were washed and resuspended in PBS with 0.1% sodium azide and measured using flow cytometry.

### MRGPRX2 Silencing by DsiRNA Electroporation

PBCMCs at a concentration of 1x10^6^ cells/mL were washed twice in cold serum-free Opti-MEM I medium (Gibco Invitrogen) and resuspended in 200 µL of the same medium. Cells were transferred to a 4.0-mm electroporation cuvette (Cell Projects), and 1 µM pool of two DsiRNA against *MRGPRX2* at a 1:1 ratio or a non–targeting control DsiRNA (Integrated DNA Technologies, Catalog #:51-01-14-03) were added to the cuvette (Duplex sequences: DsiRNA 1: 5’-GGCAUUCAGUGGUUCCUAAUAUUAT-3’ and 3’-AACCGUAAGUCACCAAGGA UUAUAAUA-5’, DsiRNA 2: 5’GUUACGUGUUCCA CAGAAUAAAATA-3’ and 3’-UUCA AUGCACAAGGUGUCUUAUUUUAU-5’). A square wave protocol (500 V, 5 ms, 0 gap, 1 pulse) was used to electroporate the cells (Gene Pulser Xcell™ device, Bio-Rad Laboratories). Immediately after electroporation, cells were transferred to 5 mL of IMDM medium (Thermofischer Scientific) supplemented with 10% fetal bovine serum (FBS, Sigma-Aldrich) (pre-heated at 37°C) and incubated for 20 min at 37°C and 5% CO_2_. Thereafter, cells were centrifuged and transferred to IMDM medium with SCF and IL-6. Five days after electroporation, a repeated analysis of MRGPRX2 expression and functionality of the PBCMCs was performed as described above.

### Sugammadex-Atracurium Inclusion Complex (S-A-Cx) Experiments

To investigate the effects of sugammadex on MRGPRX2-mediated activation of MCs induced by atracurium, cells were stimulated with atracurium and sugammadex (Bridion^®^; MSD), alone or together (designated as S-A-Cx 1, 2 and 3), in equimolar concentrations (atracurium: 538 µM, 1076 µM and 2152 µM; sugammadex: 551 µM, 1102 µM and 2204 µM). S-A-Cx 1 consisted of 538 µM atracurium and 551 µM sugammadex, S-A-Cx 2 of 1076 µM atracurium and 1102 µM sugammadex, and S-A-Cx 3 of 2152 µM atracurium and 2204 µM sugammadex. Based on the known affinity of sugammadex for atracurium, we estimated the remaining free atracurium concentrations of S-A-Cx 1, 2, and 3 to be 263 µM, 407 µM, and 612 µM, respectively. Therefore, outcomes from the S-A-Cx analyses were compared with results obtained in cells exposed to the corresponding free atracurium concentration. The activation and degranulation were studied using both intracellular calcium staining and CD63-upregulation, according to the protocol described above.

### *In Vivo* and *Ex Vivo* Testing for Responses to Rocuronium

We assessed eight patients with previous IDHRs and rocuronium hypersensitivity was documented by skin testing, as previously described (19). Their PBCMCs were investigated for MRGPRX2 levels and function. Skin tests were done with rocuronium (Esmeron^®^; Merck Sharp and Dohme, Brussels, Belgium), saline buffer as a negative control and histamine as a positive control (10 mg/mL; HAL Allergy Benelux NV, Haarlem, The Netherlands), first as skin prick tests (SPTs) and, if negative, by intradermal tests (IDTs). The maximal non-irritating rocuronium concentration was 10 mg/mL for SPTs and 0.05 mg/mL for IDTs. Rocuronium was diluted immediately before use. SPTs with a wheal ≥ 3 mm with surrounding erythema after 15 minutes were considered positive. For IDTs, injection of 0.02 mL was performed, and reactions were read after 20-30 minutes. IDT responses with a wheal surrounded by an erythema ≥ 8 mm (or doubling compared to injection bleb) were considered positive. The direct basophil activation test (BAT) for rocuronium using the patients’ basophils was performed as described (20). Results were expressed as the net percentages of CD63^+^ basophils, and the threshold of positivity was set at 4% (20). Total IgE and specific IgE (sIgE) to morphine were quantified by the FEIA ImmunoCAP system (Phadia Thermo Fisher, Uppsala, Sweden) according to the manufacturer’s instructions (21). As shown in **Table 1**, patients were stratified by the results of their rocuronium basophil test and/or levels of specific IgE as positive (i.e. IgE-dependent, patients 1-4) and negative (i.e. non IgE-dependent and probably MRGPRX2-mediated, patients 5-8). MRGPRX2 expression and functionality of their PBCMCs were compared between the two groups (16).

**Table 1:** Outcome of rocuronium skin and basophil activation tests as well as levels of rocuronium-specific IgE in patients with immediate hypersensitivity reactions to rocuronium.

Patient	Rocuronium response assessed			Total IgE
	Skin test positivity	BAT	sIgE*	
1	SPT	pos	neg/0.5	46.5
2	SPT	pos	0.3/3.2	58
3	IDT (0.01 mg/mL)	NR	0.4/2.1	157
4	IDT (0.05 mg/mL)	NR	10/92.7	1118
5	SPT	neg	neg	213
6	IDT (0.05 mg/mL)	neg	neg	193
7	IDT (0.05 mg/mL)	neg	neg	26
8	IDT (0.05 mg/mL)	neg	neg	455

Skin tests were done with rocuronium (10 mg/mL), saline buffer as a negative control and histamine as a positive control (10 mg/mL), first as skin prick tests and, if negative, by intradermal tests. SPT, skin prick test; IDT, intradermal test; BAT, basophil activation test; sIgE, specific IgE; pos, positive test; neg, negative test; NR, non-responder; total IgE is expressed as kU/L; ^*^ data provided are for specific IgE, in kUA/L, to rocuronium/morphine, with thresholds for positivity: > 0.13 kUA/L (21)/>0.35 kUA/L. Specific IgE to morphine is a marker for sensitization to tertiary and quaternary ammonium structures (21–23).

PBCMCs of rocuronium-hypersensitive patients were functionally studied using intracellular calcium staining and CD63 upregulation as described above. PBCMCs were stimulated with tyrode buffer, substance P (74 µM), anti-FcϵRI (0.5, 2.5 and 5.0 µg/mL), amoxicillin (1370 µM, GSK), morphine hydrochloride (50 µM, 250 µM and 500 µM, Sterop), or rocuronium bromide (1.64 µM, 16.4 µM, 164 µmM, 1640 µM; Esmeron^®^, Organon). Dose response testing was performed as previously described (17).

### Flow Cytometric Analyses

Flow cytometric analyses were performed on a calibrated FACSCalibur (BD Immunocytometry systems) with argon-ion lasers (488nm and 633nm), for the intracellular calcium measurements, or on a calibrated FACSCanto II flow cytometer (BD Immunocytometry Systems, San Jose, CA) equipped with three lasers (405 nm, 488 nm and 633 nm), for the CD63 measurement. Correct compensation settings were performed using BD CompBeads (BD Biosciences). Flow cytometric data were analyzed using Kaluza Analysis 2.1 software (Beckman Coulter) and FCS6 Express 6 flow research edition (*de novo* software, Glendale, California). A fluorescence minus one (FMO) sample was used to set a marker between positive and negative cells according to the 99^th^ percentile. The results of the calcium measurements were expressed as fold increase against the basal intensity. Results of CD63 measurements were expressed as the net value of percentages of positive cells, i.e. the percentage of CD63^+^ cells in stimulated cells minus the percentage of CD63^+^ cells in resting cells. Al least 500 PBCMCs were counted per sample.

### Statistical Analysis

GraphPad Prism version 7 software was used for data analysis and paired Student’s t-tests were performed. Results are expressed as mean ± SEM. A P-value of < 0.05 was considered significant. The “n” in the figures denotes the total number of different donors used.

### Ethical Considerations

Our study was approved by the Ethical Committee of the Antwerp University Hospital (Belgium B300201837509). All participants gave written informed consent.

## Results

### Ciprofloxacin, Levofloxacin, and Atracurium, but Not Succinylcholine, Induce Activation and Degranulation of PBCMCs *via* MRGPRX2

**Figure 1:**
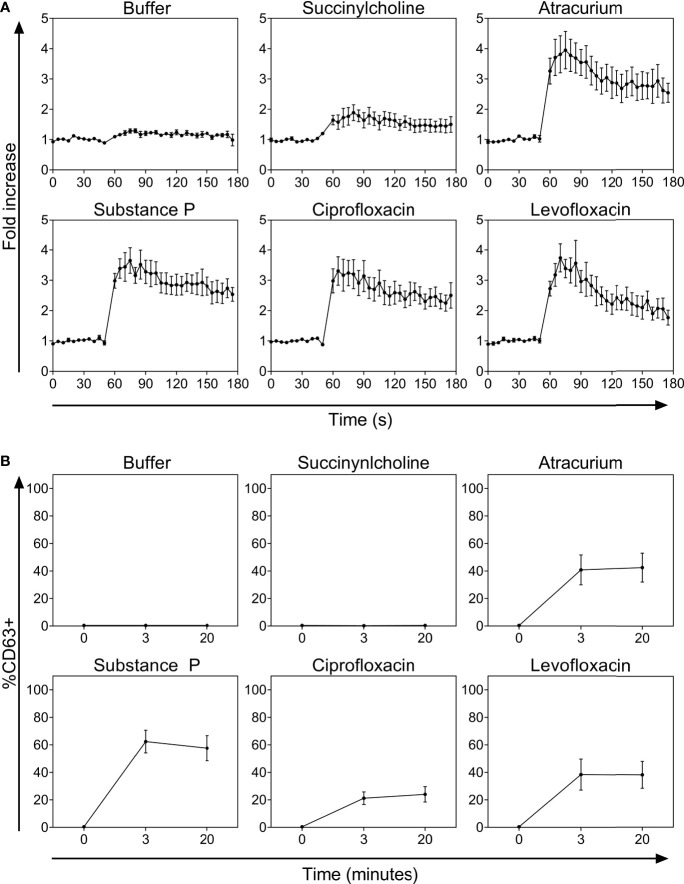
**(A)** Calcium staining and **(B)** time curves of CD63 up-regulation after incubation with buffer, the natural ligand of MRGPRX2 substance P (74 µM), succinylcholine (5536 µM), atracurium (2152 µM), ciprofloxacin (755 µM) or levofloxacin (2767 µM). In all experiments, n=5. Fold increase is calculated against the basal intensity. s, seconds.

The FQs ciprofloxacin and levofloxacin potently activated PBCMCs, with a rapid increase in intracellular calcium (**Figure 1A** and **Supplementary Figure 3**). They also induced degranulation, with upregulated surface expression of the lysosomal degranulation marker CD63 (**Figure 1B**). Of the two NMBAs tested, atracurium, but not succinylcholine, induced significant activation of PBCMCs as demonstrated by increase in intracellular calcium (**Figure 1A** and **Supplementary Figure 3**), and degranulation, i.e. upregulation of CD63 (**Figure 1B**). EC50 values are displayed in **Table 2**.

**Table 2:** EC50 values.

	EC50 (µM)
**Atracurium**	391 µM
**Ciprofloxacin**	319 µM
**Levofloxacin**	1557 µM

**Figure 2:**
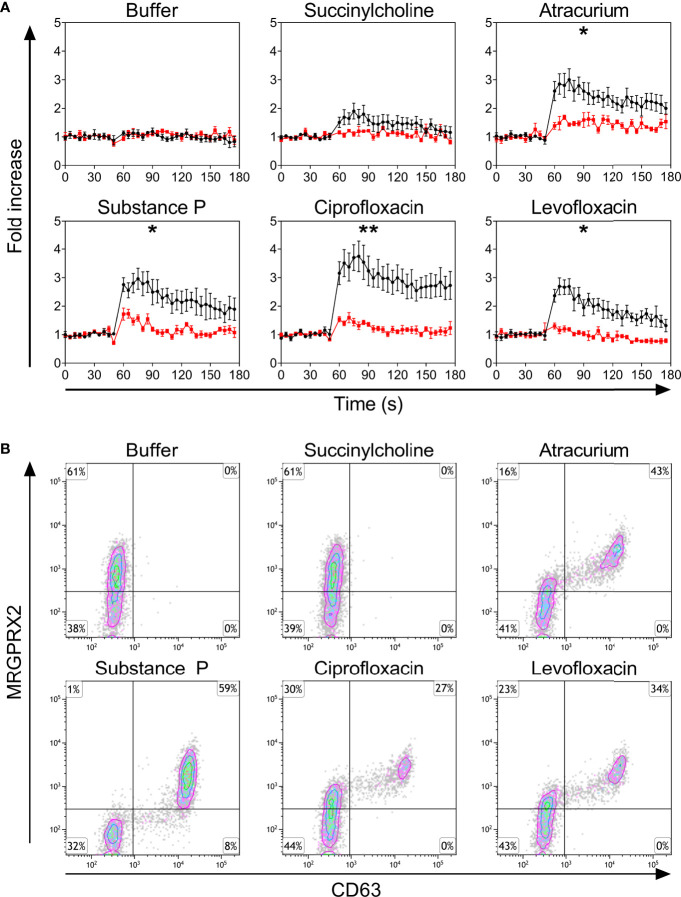
**(A)** Calcium imaging in the MRGPRX2^+^ (black) and MRGPRX2^-^ (red) subpopulations and **(B)** up-regulation of surface CD63. **(A)** Calcium staining and **(B)** representative plots for CD63 up-regulation after incubation with buffer, the natural ligand of MRGPRX2 substance P (74 µM), succinylcholine (5536 µM), atracurium (2152 µM), ciprofloxacin (755 µM) or levofloxacin (2767 µM). For the representative plot, PBCMCs were incubated for 3 min. In all experiments, n=5. Fold increase is calculated against the basal intensity. Area under the curves were compared using a paired student t-test, p < 0.05*, p < 0.01**. s, seconds.

**Figure 3:**
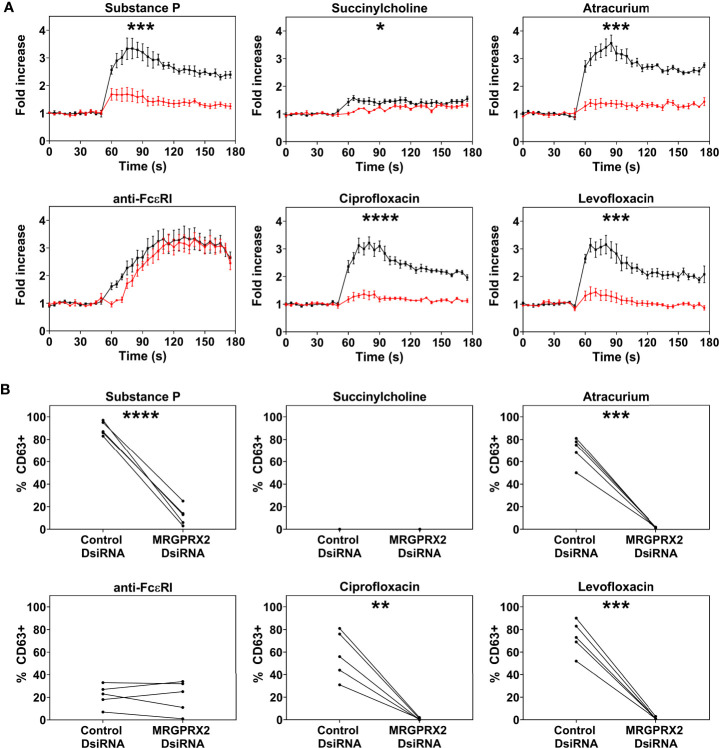
Effect of MRGPRX2 silencing on PBCMC functionality after *MRGPRX2*-specific DsiRNA electroporation. PBCMCs were electroporated with a negative control DsiRNA (black) or a *MRGPRX2*-specific DsiRNA (red). **(A)** Effect of the silencing on the calcium levels and **(B)** CD63 up-regulation after 3 min of incubation. Incubation with substance P (74 µM), the natural agonist of MRGPRX2, anti-FcϵRI (2.5 µg/mL), succinylcholine (5536 µM), atracurium (2152 µM), ciprofloxacin (755 µM) or levofloxacin (2767 µM). In all experiments, n=5. Area under the curves were compared using a paired student t-test, p < 0.05*, p < 0.01**, p < 0.001***, p < 0.0001****. Note that the effect of MRGPRX2 silencing align with the differences observed between MRGPRX2^+^ and MRGPRX2^-^ cells as shown in **Figure 1B** and **Supplementary Figure 4**. As shown previously, MRGPRX2 silencing has no significant effect on anti-FcϵRI-dependent intracellular calcium signalling and degranulation (17). s, seconds.

Only PBCMCs that expressed MRGPRX2, but not MRGPRX2-negative PBCMCs, showed significant activation by ciprofloxacin, levofloxacin, and atracurium, as assessed by calcium flux (**Figure 2A** and **Supplementary Figure 4**) as well as degranulation (**Figure 2B**). Moreover, degranulation of MRGPRX2-positive PBCMCs was most pronounced in cells with the highest surface expression of MRGPRX2 (**Figure 2B**). Silencing of MRGPRX2 in PBCMCs, by treatment with MRGPRX2-DsiRNA, reduced MRGPRX2 expression by more than 70% and prevented PBCMCs activation and degranulation by ciprofloxacin, levofloxacin, and atracurium (**Figure 3** and **Supplementary Figures 5**–**7**).

### Sugammadex Attenuates Atracurium-Induced Activation and Degranulation of PBCMCs

**Figure 4:**
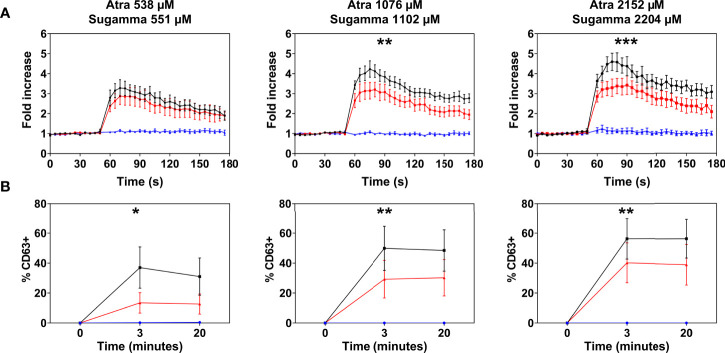
Effect of sugammadex on PBCMC activation induced by atracurium. **(A)** Intracellular calcium staining or **(B)** CD63 upregulation of PBCMCs after incubation with atracurium (538 µM, 1076 µM or 2152 µM) (black), sugammadex (551 µM, 1102 µM or 2204 µM) (blue) or the S-A-Cx in equimolar concentrations (red). In all experiments, n=5. Area under the curves were compared using a paired student t-test, p < 0.05*, p < 0.01**, p < 0.001***, p < 0.0001***. s, seconds.

**Figure 5:**
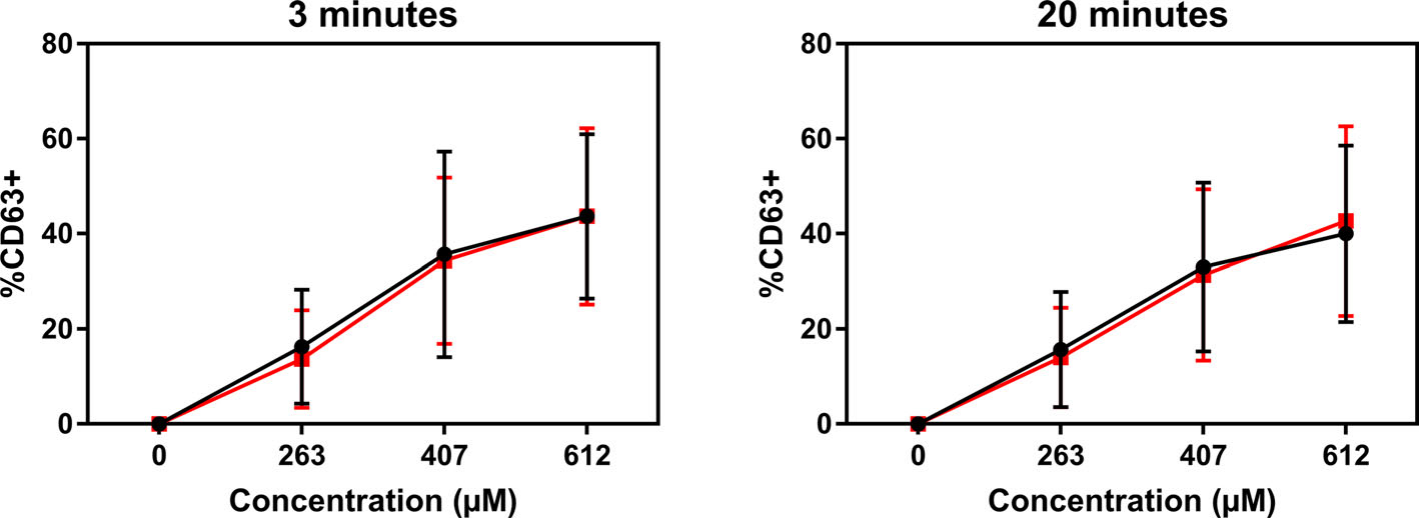
Sugammadex-atracurium complexes (S-A-Cx) and corresponding free atracurium show similar mast cell degranulation. CD63 upregulation of PBCMCs after incubation for 3 or 20 minutes with the S-A-Cx (red) or the corresponding estimated concentrations of free atracurium (black) (263 µM, 407 µM or 612 µM). In all experiments, n=3.

Atracurium dose-dependently activated and degranulated PBCMCs, *via* MRGPRX2, whereas sugammadex did not cause PBCMC activation or degranulation (**Figure 4** and **Supplementary Figure 4**). Complexing of atracurium with sugammadex, at a 1:1 molar ratio, markedly reduced, but did not completely eliminate the activation and degranulation of PBCMCs (**Figure 4**). Degranulation by free atracurium, induced at the concentrations achieved by complexing with sugammadex, was comparable to that induced by the sugammadex-atracurium complexes (**Figure 5**).

### Rocuronium Causes Similar Activation and Degranulation of PBCMCs From Rocuronium-Hypersensitive Patients Who Have Specific IgE and Those Who *Do Not*

**Figure 6:**
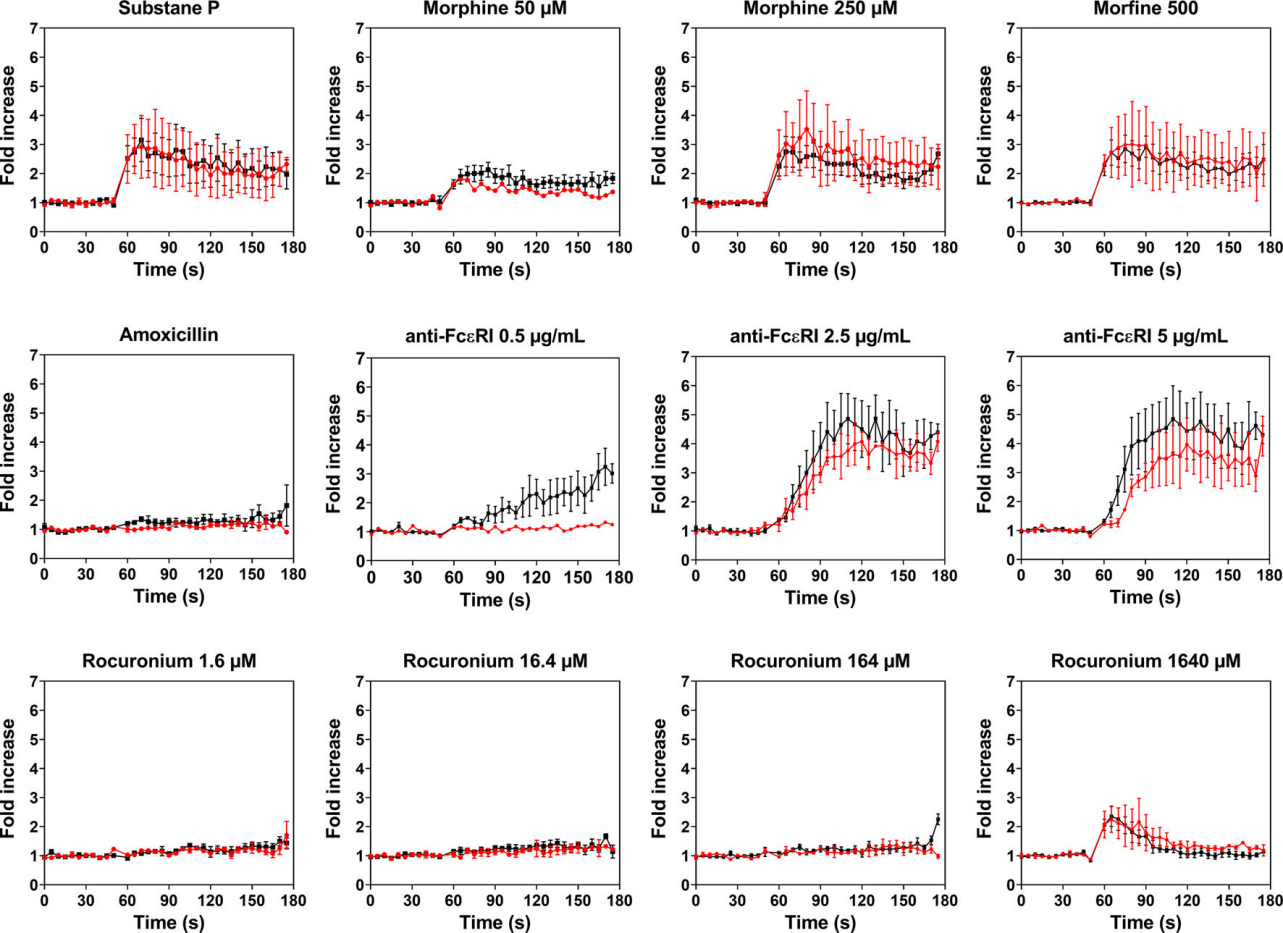
Calcium imaging of PBCMCs derived from patients with IgE-mediated (red) and non IgE-mediated, i.e. probably MRGPRX2-mediated, rocuronium hypersensitivity (black). Incubation with buffer, the positive control substance P (74 µM), the natural agonist of MRGPRX2, anti-FcϵRI (0.5 µg/mL, 2.5 µg/mL or 5 µg/mL), amoxicillin (1370 µM), morphine (50 µM, 250 µM or 500 µM), or rocuronium (1.6 µM, 16.4 µM, 164 µM or 1640 µM). Fold increase is calculated against the basal intensity. N= 2 or 4. s, seconds.

**Figure 7:**
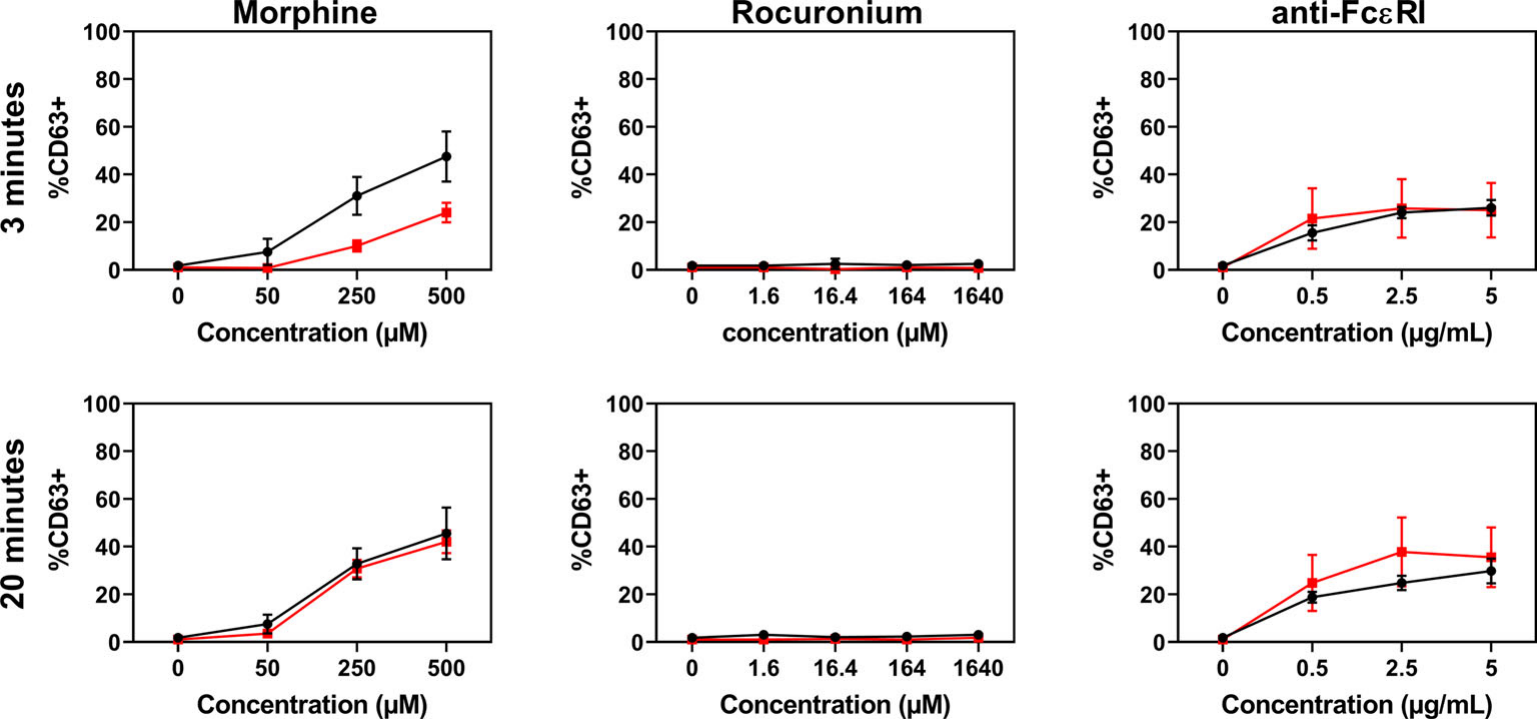
Dose-response curves for CD63 expression in PBCMCs of patients with IgE-mediated (red) and non IgE-mediated, i.e. probably MRGPRX2-mediated, rocuronium hypersensitivity (black). Dose-response curves of CD63 up-regulation after 3 min of stimulation or after 20 min of stimulation. N=4.

PBCMCs obtained from patients with rocuronium hypersensitivity, i.e. a history of rocuronium-induced IDHR, showed similar levels of activation and degranulation in patients who had specific IgE and/or a positive BAT to rocuronium as compared to those who did not (**Figures 6** and **7**). Rocuronium-hypersensitive patients with and without specific IgE and/or a positive BAT to rocuronium had similar rates of MRGPRX2-positive cells, 65 ± 11% and 63 ± 4%, respectively (**Supplementary Figure 8**).

## Discussion

Here, we answer three important questions on the role and relevance of the MRGPRX2 pathway in MC-dependent immediate hypersensitivity reactions to NMBAs and FQs (11, 15, 24, 25). We show 1) that atracurium, ciprofloxacin and levofloxacin degranulate human MCs *via* MRGPRX2, 2) that sugammadex attenuates atracurium-induced and MRGPRX2-mediated MC degranulation by reducing free atracurium, and 3) that rocuronium-hypersensitive patients with and without sensitization to this NMBA show similar mast cell MRGPRX2 expression and function. These findings help our understanding and may improve the prevention of anaphylactic reactions to NMBAs and FQs.

Our results of studies with primary human MCs show that degranulation in response to atracurium, ciprofloxacin and levofloxacin is restricted to the MRGPRX2^+^ subpopulation and that these responses can be effectively mitigated by silencing of MRGPRX2. In contrast, succinylcholine did not degranulate human MCs. These observations are in line with the results of previous studies with murine cells and human MC lines (11, 14, 25, 26). Our results also confirm that selective MRGPRX2 silencing in primary human MCs *via* electroporation of DsiRNA against *MRGPRX2* enables studying the effects of receptor activation on both calcium mobilization and degranulation (17). As shown previously, this silencing approach has a high transfection efficiency and results in markedly downregulated cell surface levels of MRGPRX2 (17). Combined with the novel flow cytometric technique we used in this study, this approach enables analyses at the single cell level, which allows for the identification and characterization of cellular subsets in non-homogeneous populations, such as PBCMCs with MRGPRX2 positive and negative subsets. This also explains why traditional mediator release tests, which only provide averaged results across all cells, are of limited use for the characterization of MRGPRX2-mediated responses in human MCs.

How does sugammadex, a NMBA reversal agent, prevent atracurium-induced and MRGPRX2-induced adverse reactions? Earlier work with leukemic LAD-2 cells demonstrated that co-incubation of atracurium with sugammadex, in molar excess, can inhibit atracurium-induced MRGPRX2-mediated calcium mobilization and degranulation (14). Our findings from studies with primary human PBCMCs confirm that this is indeed the case. More importantly, our study shows that sugammadex reduces MRGPRX2 responses by sequestration of free atracurium. Sugammadex-atracurium complexes, by themselves, are not inhibitory. While this is interesting, mechanistically, it may be of limited clinical relevance, as recent reports suggest that sugammadex does not stop atracurium-induced basophil and MC degranulation, once initiated (14, 19). This argues against the use of sugammadex in patients with ongoing atracurium-mediated perioperative anaphylaxis (27).

That NMBAs induced MRGPRX2-related IDHRs occur only in some patients and not most or all has been suggested to be due to higher levels of MRGPRX2 expression or susceptibility to activation in the former (10, 15). Our findings suggest that this is probably not the case. The mast cells of rocuronium-hypersensitive patients without specific IgE to rocuronium and a negative BAT, i.e. patients with IgE-independent IDHRs to rocuronium, had the same levels of MRGPRX2 expression and the same susceptibility to MRGPRX2 activation as rocuronium-sensitized patients, i.e. patients with IgE-dependent IDHRs to rocuronium (16). Further studies aimed at identifying those at risk of rocuronium IDHRs should focus on alternative mechanisms (15). Our results come with two caveats. First, we analyzed patient PBCMCs rather than skin mast cells (there are some differences) (10), and second, our conclusions are based on the assumption that positive sIgE and BAT results are indicative for an IgE-dependent reaction and that the absence of sIgE and a negative BAT point to a MRGPRX2-dependent reaction. There is, currently, no validated assay to test patients for MRGPRX2-dependent anaphylaxis. Exclusion of other mechanisms, i.e. IgE-mediated activation, by sIgE and BAT is the best approach to identifying patients with probably MRGPRX2-dependent reactions (16, 28). Unlike MCs, resting circulation basophils barely express MRGPRX2 (29, 30) and have repeatedly been shown not to respond in a non-specific manner to potent MRGPRX2 agonists such as opiates (e.g. morphine, codeine and pholcodine) (31, 32), FQs (e.g. moxifloxacin, ciprofloxacin, levofloxacin) (6, 33) and certain NMBAs (e.g. atracurium, mivacurium) (3, 34, 35). Positive CD63-BAT results together with sIgE and wortmannin inhibition experiments point to patients with specific sensitization and IgE-dependent reactions (32, 33, 36).

Our study has several strengths and limitations. Its major strength is that we assessed MRGPRX2, across all three questions we addressed, with novel and powerful models and techniques including primary human MCs generated from peripheral blood, silencing of MRGPRX2 *via* DsiRNA electroporation, and single cell flow cytometric analyses. Its limitations include the relatively low number of patients analyzed, which did not allow for meaningful comparisons of subpopulations of patients, owed largely to the rare occurrence of NMBA-mediated IDHRs. Another limitation of our approach, as in many *in vitro* and *ex vivo* experiments, lies in the necessity of supra-therapeutic (sometimes near toxic) stimulation concentrations.

Taken together, this is the first study to show, in primary human MCs, that the NMBA atracurium as well as the FQs ciprofloxacin and levofloxacin induce degranulation *via* MRGPRX2, that sugammadex attenuates atracurium-induced MRGPRX2-activation and downstream MC degranulation by reducing free atracurium, and that MCs of rocuronium-hypersensitive patients with IgE-independent IDHRs to this NMBA are not different in their MRGPRX2 expression and function as compared to patients with IgE-dependent IDHRs. We also conclude that the use of PBCMCs together with *MRGPRX2*-targeted silencing can contribute to answer further questions on the role and relevance of MRGPRX2 in IDHRs and beyond (37). Our results should encourage such studies, as a better understanding of the MRGPRX2 pathway is needed to improve the prevention and treatment of anaphylactic reactions to NMBAs and FQs.

## Data Availability Statement

The raw data supporting the conclusion of this article are available on request from the corresponding author.

## Ethics Statement

Our study was approved by the Ethical Committee of the Antwerp University Hospital (Belgium B300201837509). All participants gave written informed consent. The patients/participants provided their written informed consent to participate in this study. Written informed consent was obtained from the individual(s) for the publication of any potentially identifiable images or data included in this article.

## Author Contributions

JE performed all experiments and wrote the paper. The experiments were performed under supervision of CB, CM and MH who also contributed to the experimental design. The electroporation experiments were performed under supervision of VT, EL and DC-D. VS and DE coordinated and supervised the project and wrote the paper. All authors contributed to the article and approved the submitted version.

## Conflict of Interest

MM has received honoraria (advisory board, speaker) and/or institutional grant/research support from Allakos, Amgen, Astra-Zeneca, Bayer, Dr. Pfleger, FAES, Genentech, GSK, Innate Pharma, Kyowa Kirin, Lilly, Merckle Recordati, Moxie, Novartis, Regeneron, Roche, Sanofi, MSD, UCB, and Uriach.

The remaining authors declare that the research was conducted in the absence of any commercial or financial relationships that could be construed as a potential conflict of interest.

## Publisher’s Note

All claims expressed in this article are solely those of the authors and do not necessarily represent those of their affiliated organizations, or those of the publisher, the editors and the reviewers. Any product that may be evaluated in this article, or claim that may be made by its manufacturer, is not guaranteed or endorsed by the publisher.

## Supplementary Material

Supplementary Figure 1 Representative plot for the MRGPRX2 expression on PBCMCs. Peripheral blood cultured mast cells (PBCMCs) are defined as CD117^+^CD203c^+^ cells. PBCMCs harbor two subpopulations: cells with surface expression of MRGPRX2 (MRGPRX2^+^) and cells without expression of MRGPRX2 (MRGPRX2^-^).

Supplementary Figure 2 Dose-response curves for changes in intracellular calcium and CD63 expression in PBCMCs. **(A)** Dose-response curves of intracellular calcium levels. **(B)** Dose-response curves of CD63 up-regulation after 3 min of stimulation or **(C)** after 20 min of stimulation. Attempts to increase the ciprofloxacin concentration revealed to be cytotoxic.

Supplementary Figure 3 Representative plots for intracellular calcium imaging in PBCMCs. PBCMCs were, after 50 sec, stimulated with buffer, substance P (74 µM), the natural agonist of MRGPRX2, succinylcholine (5536 µM), atracurium (2152 µM), ciprofloxacin (755 µM) or levofloxacin (2767 µM).

Supplementary Figure 4 Representative plot for intracellular calcium imaging in MRGPRX2^+^ **(A)** and MRGPRX2^-^ **(B)** subpopulations. PBCMCs were, after 50 sec, stimulated with buffer, substance P (74 µM), the natural agonist of MRGPRX2, succinylcholine (5536 µM), atracurium (2152 µM), ciprofloxacin (755 µM) or levofloxacin (2767 µM).

Supplementary Figure 5 Silencing of the MRGPRX2-receptor after electroporation with *MRGPRX2*-specific DsiRNA. A comparison of the surface expression of MRGPRX2 or FcϵRI between PBCMC electroporated with control DsiRNA or DsiRNA specific for *MRGPRX2*. In all experiments, n=5. p < 0.001***. As shown earlier, the maximal expression of FcϵRI remains unaltered in MRGPRX2 silenced cells (17).

Supplementary Figure 6 Representative plots of CD63 up-regulation after silencing of *MRGPRX2*. Cells were electroporated with a negative control **(A)** or *MRGPRX2*-specific DsiRNA **(B)**. Thereafter, cells were incubated with buffer, substance P (74 µM), anti-FcϵRI (2.5 µg/mL), succinylcholine (5536 µM), atracurium (2152 µM), ciprofloxacin (755 µM) or levofloxacin (2767 µM). MRGPRX2 silencing has no significant effect on anti-FcϵRI-dependent intracellular calcium signalling (17).

Supplementary Figure 7 Representative plots of CD63 up-regulation after silencing of *MRGPRX2*. Cells were electroporated with a negative control **(A)** or target specific DsiRNA **(B)**. Thereafter, cells were incubated for 3 minutes with buffer, substance P (74 µM), anti-FcϵRI (2.5 µg/mL), succinylcholine (5536 µM), atracurium (2152 µM), ciprofloxacin (755 µM) or levofloxacin (2767 µM). MRGPRX2 silencing has no significant effect on anti-FcϵRI degranulation (17).

Supplementary Figure 8 MRGPRX2 expression on PBCMCs cultured from patients with an IgE-dependent and non IgE-mediated, i.e. probably MRGPRX2-mediated, rocuronium hypersensitivity reaction. Peripheral blood cultured mast cells (PBCMCs) are defined as CD117^+^CD203c^+^ cells. PBCMCs harbor two subpopulations: cells with surface expression of MRGPRX2 (MRGPRX2^+^) and cells without expression of MRGPRX2 (MRGPRX2^-^).

## Abbreviations

BAT, Basophil activation test; DsiRNA, dicer small interference RNA; FcϵRI, High affinity receptor for IgE; FMO, fluorescence minus one; FQ(s), fluoroquinolone(s); IDHR(s), immediate drug hypersensitivity reaction(s); IDT(s), intradermal test(s); IgE, Immunoglobulin E; IL, interleukin; LDL, low-density lipoprotein; MC(s), mast cell(s); MRGPRX2, mas-related G protein coupled receptor X2; NMBA(s), neuromuscular blocking agent(s); PBCMC(s), peripheral blood cultured mast cell(s); PBMC(s), peripheral blood mononuclear cell(s); S-A-Cx, sugammadex-atracurium inclusion complex; SCF, stem cell factor; SEM, standard error of the mean; sIgE, specific IgE antibody; SPT(s), skin prick test(s).
