# Supplementary figures and images for: Novel Insights on MRGPRX2-Mediated Hypersensitivity to Neuromuscular Blocking Agents And Fluoroquinolones
Source: Front Immunol. 2021 Jul 27;12:668962. doi: 10.3389/fimmu.2021.668962 (PMC8353374; doi:10.3389/fimmu.2021.668962)

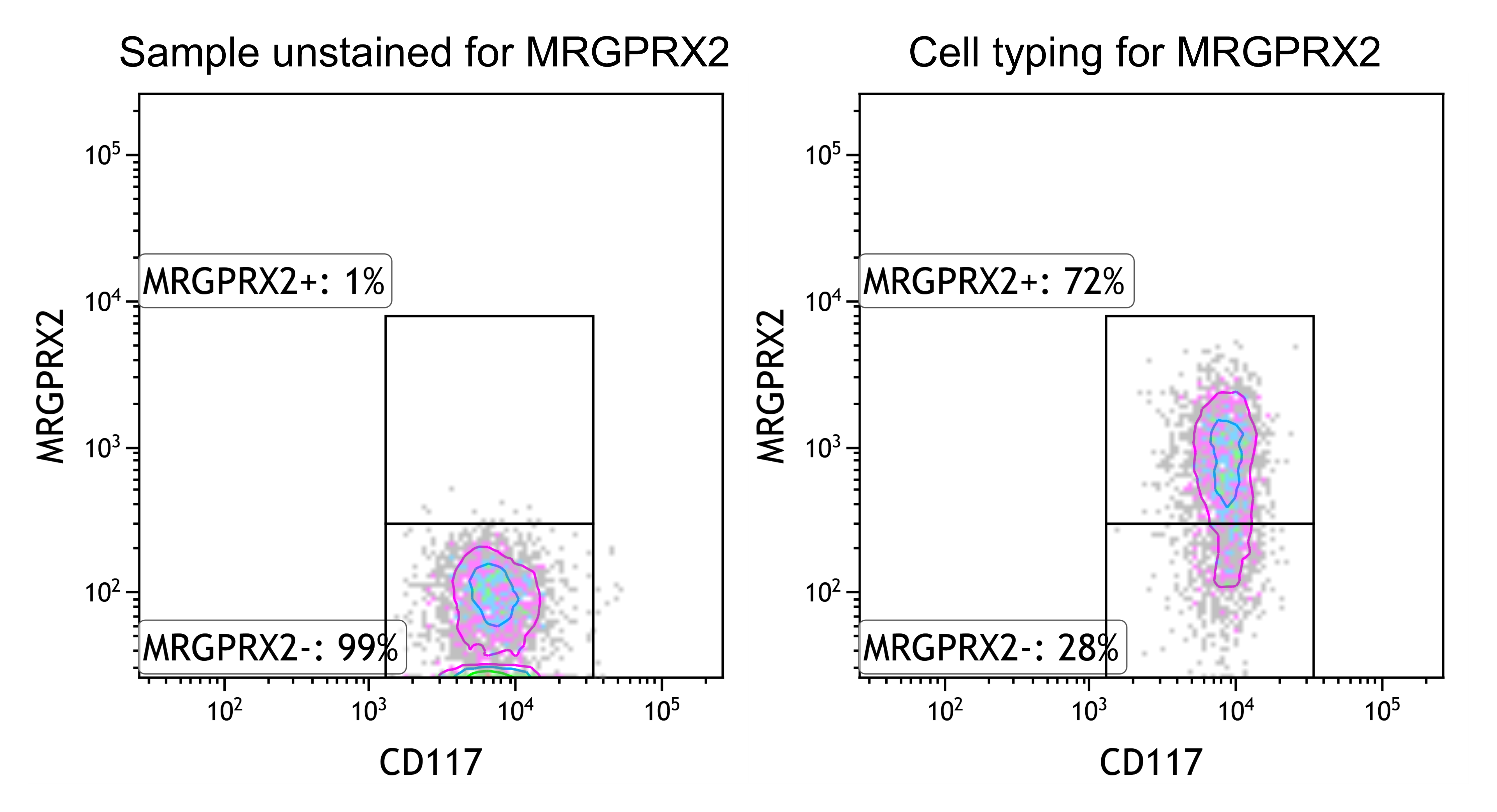

Supplement: Supplementary Figure 1 — Representative plot for the MRGPRX2 expression on PBCMCs. Peripheral blood cultured mast cells (PBCMCs) are defined as CD117+CD203c+ cells. PBCMCs harbor two subpopulations: cells with surface expression of MRGPRX2 (MRGPRX2+) and cells without expression of MRGPRX2 (MRGPRX2-). [file Image_1.tif]

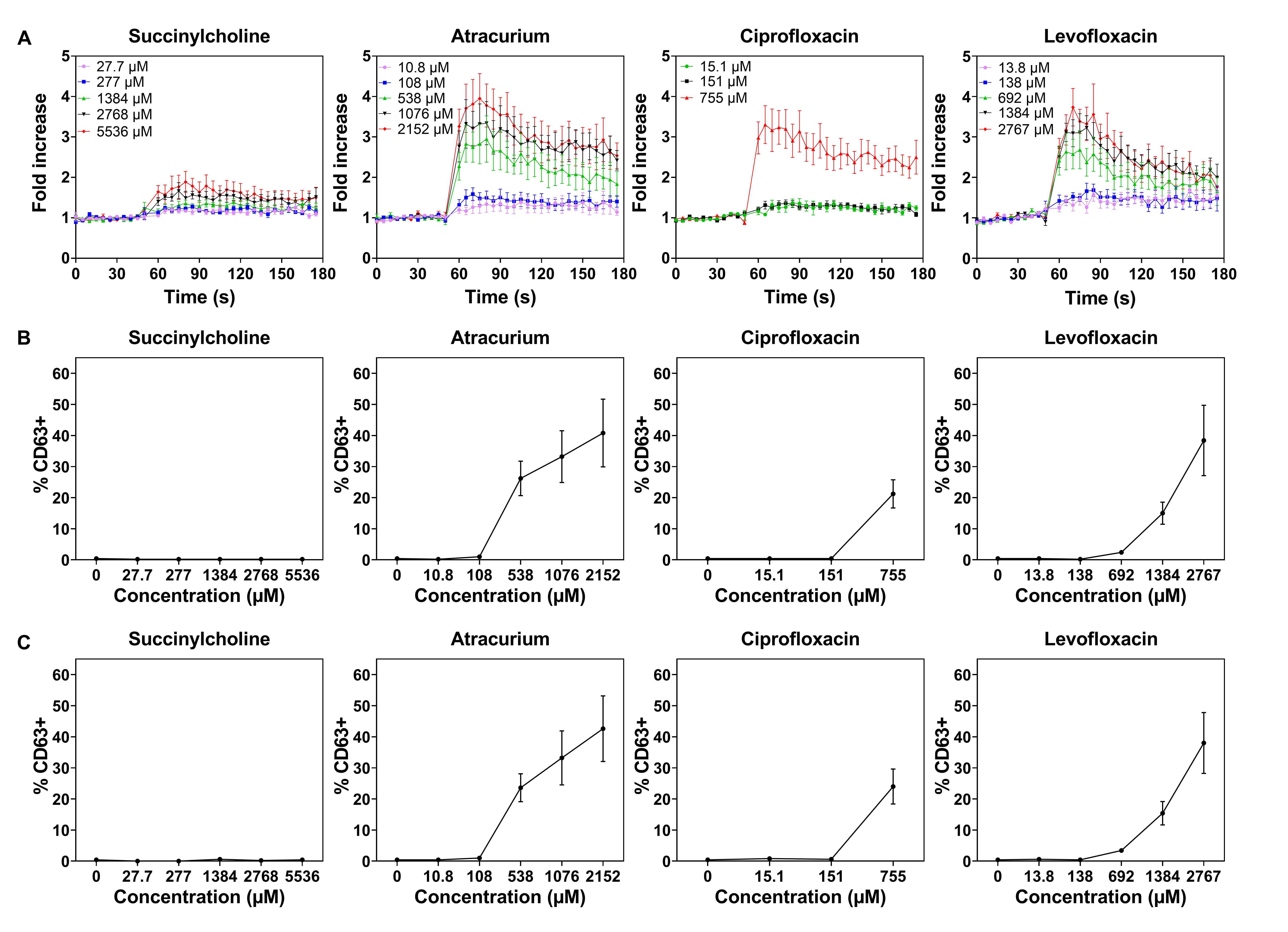

Supplement: Supplementary Figure 2 — Dose-response curves for changes in intracellular calcium and CD63 expression in PBCMCs. (A) Dose-response curves of intracellular calcium levels. (B) Dose-response curves of CD63 up-regulation after 3 min of stimulation or (C) after 20 min of stimulation. Attempts to increase the ciprofloxacin concentration revealed to be cytotoxic. [file Image_2.tif]

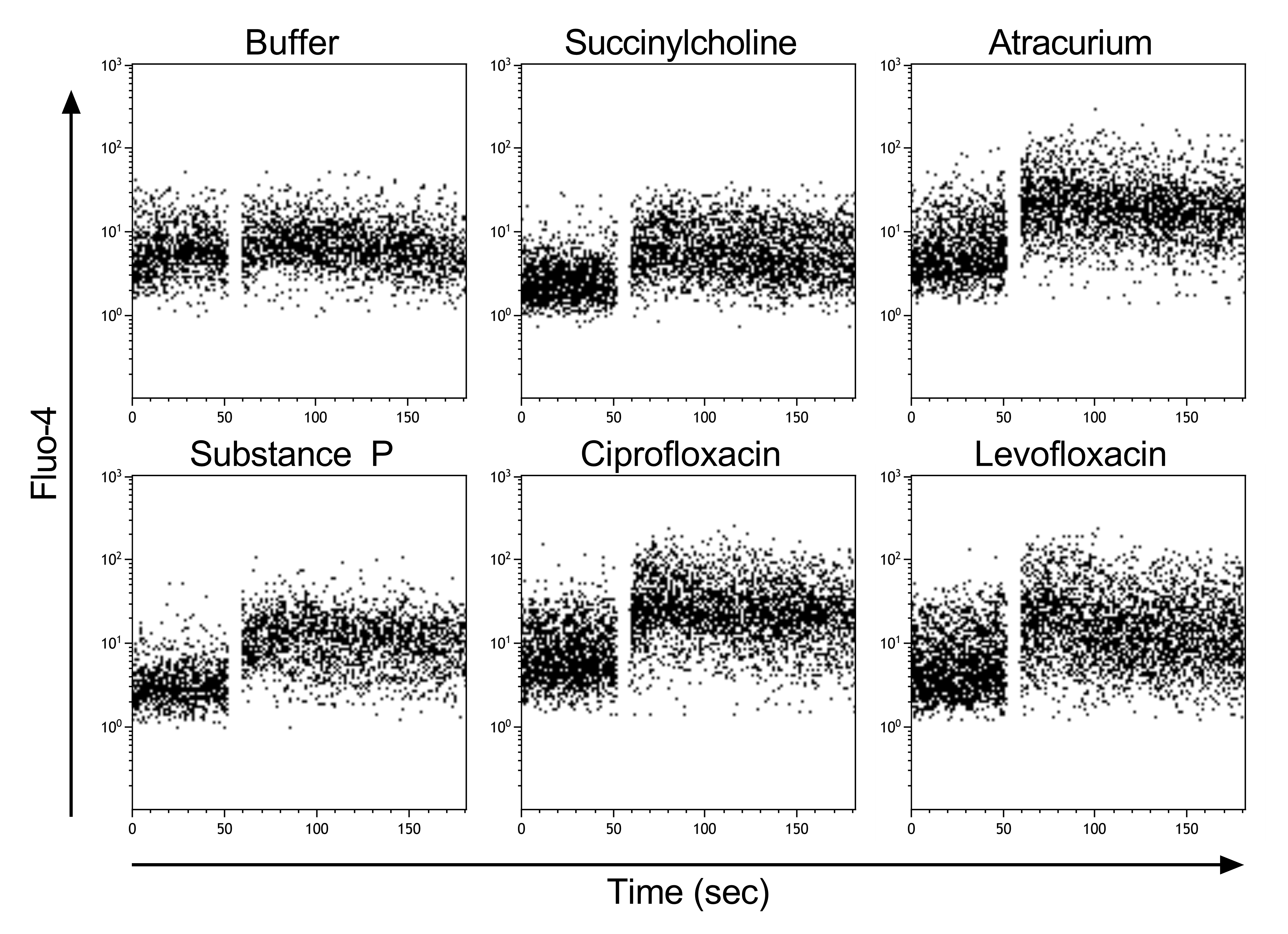

Supplement: Supplementary Figure 3 — Representative plots for intracellular calcium imaging in PBCMCs. PBCMCs were, after 50 sec, stimulated with buffer, substance P (74 µM), the natural agonist of MRGPRX2, succinylcholine (5536 µM), atracurium (2152 µM), ciprofloxacin (755 µM) or levofloxacin (2767 µM). [file Image_3.tif]

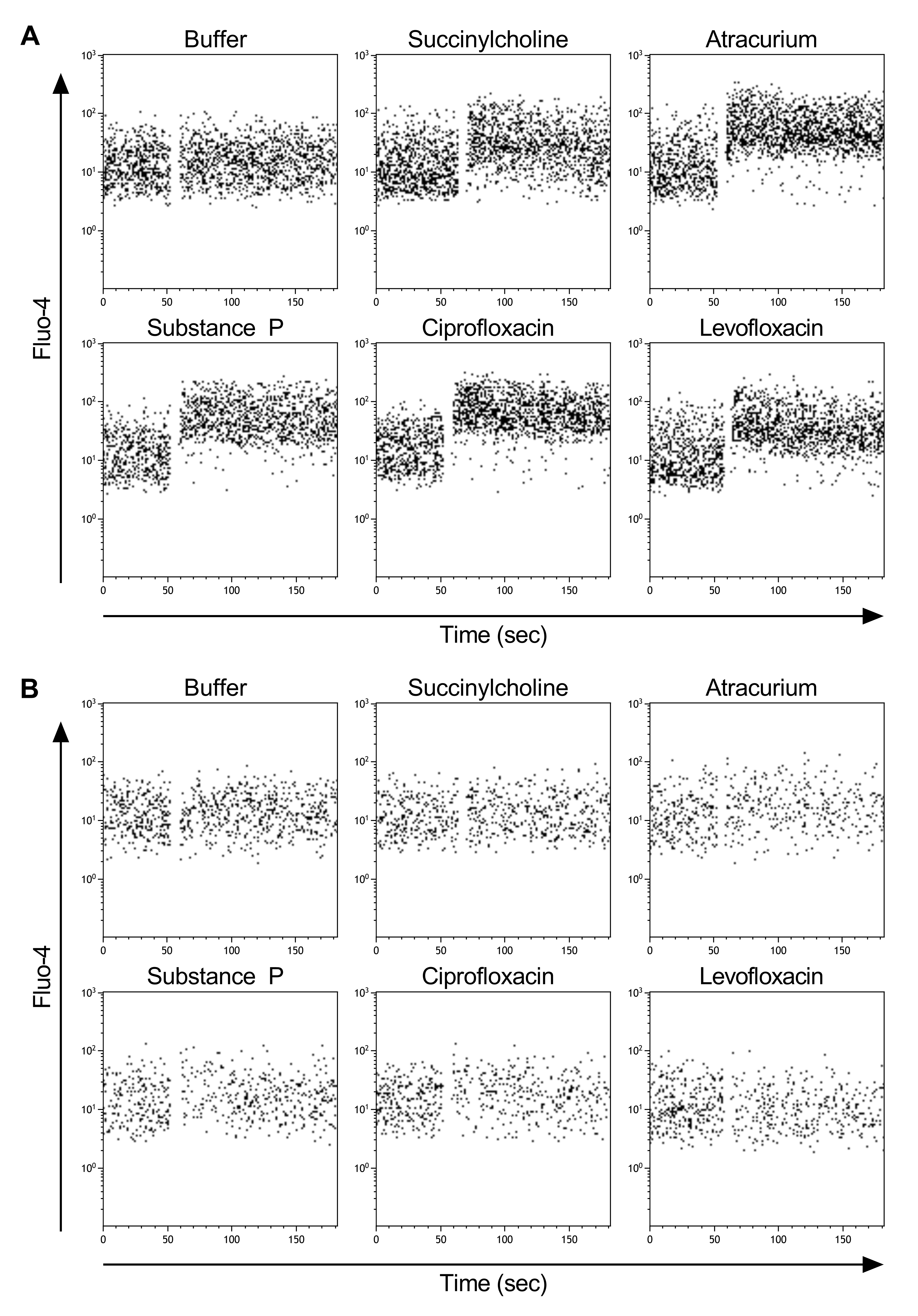

Supplement: Supplementary Figure 4 — Representative plot for intracellular calcium imaging in MRGPRX2+ (A) and MRGPRX2- (B) subpopulations. PBCMCs were, after 50 sec, stimulated with buffer, substance P (74 µM), the natural agonist of MRGPRX2, succinylcholine (5536 µM), atracurium (2152 µM), ciprofloxacin (755 µM) or levofloxacin (2767 µM). [file Image_4.tif]

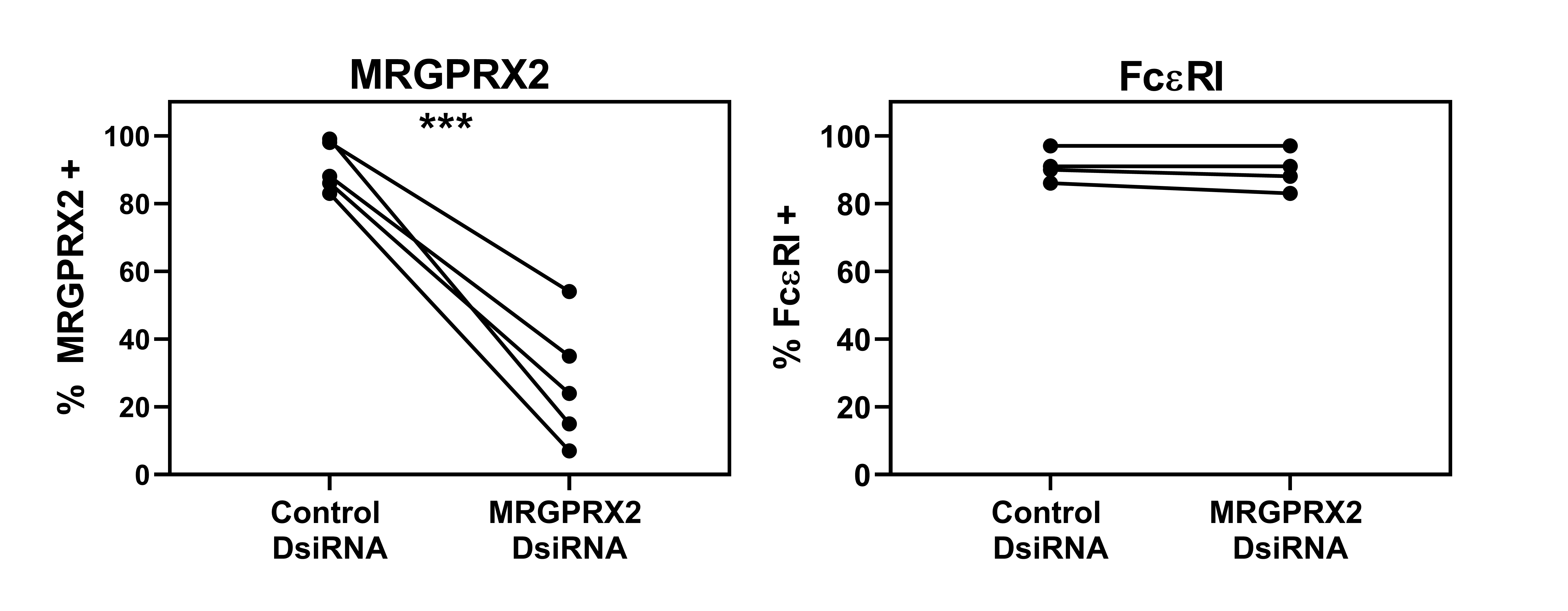

Supplement: Supplementary Figure 5 — Silencing of the MRGPRX2-receptor after electroporation with MRGPRX2-specific DsiRNA. A comparison of the surface expression of MRGPRX2 or FcϵRI between PBCMC electroporated with control DsiRNA or DsiRNA specific for MRGPRX2. In all experiments, n=5. p < 0.001***. As shown earlier, the maximal expression of FcϵRI remains unaltered in MRGPRX2 silenced cells (17). [file Image_5.tif]

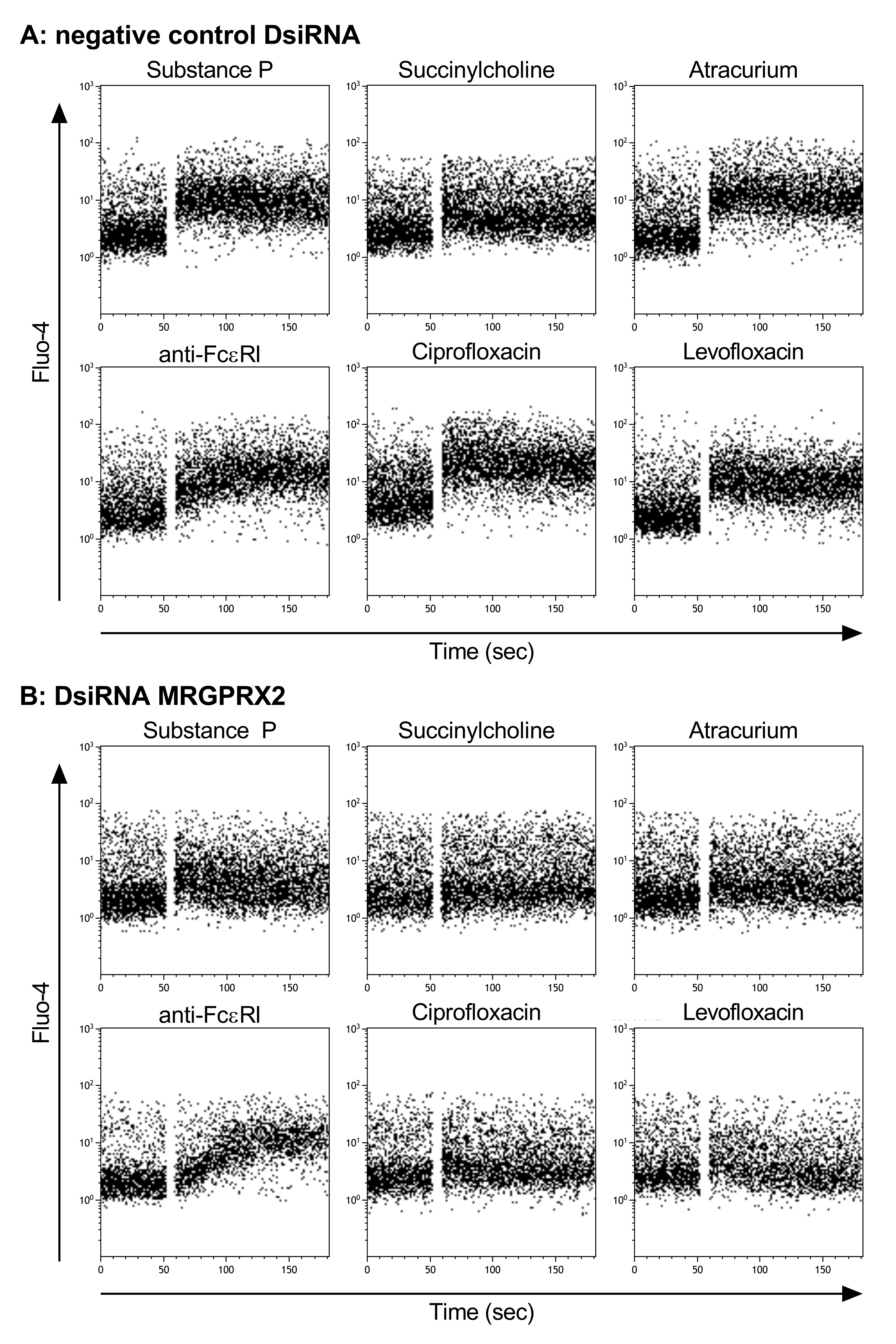

Supplement: Supplementary Figure 6 — Representative plots of CD63 up-regulation after silencing of MRGPRX2. Cells were electroporated with a negative control (A) or MRGPRX2-specific DsiRNA (B). Thereafter, cells were incubated with buffer, substance P (74 µM), anti-FcϵRI (2.5 µg/mL), succinylcholine (5536 µM), atracurium (2152 µM), ciprofloxacin (755 µM) or levofloxacin (2767 µM). MRGPRX2 silencing has no significant effect on anti-FcϵRI-dependent intracellular calcium signalling (17). [file Image_6.tif]

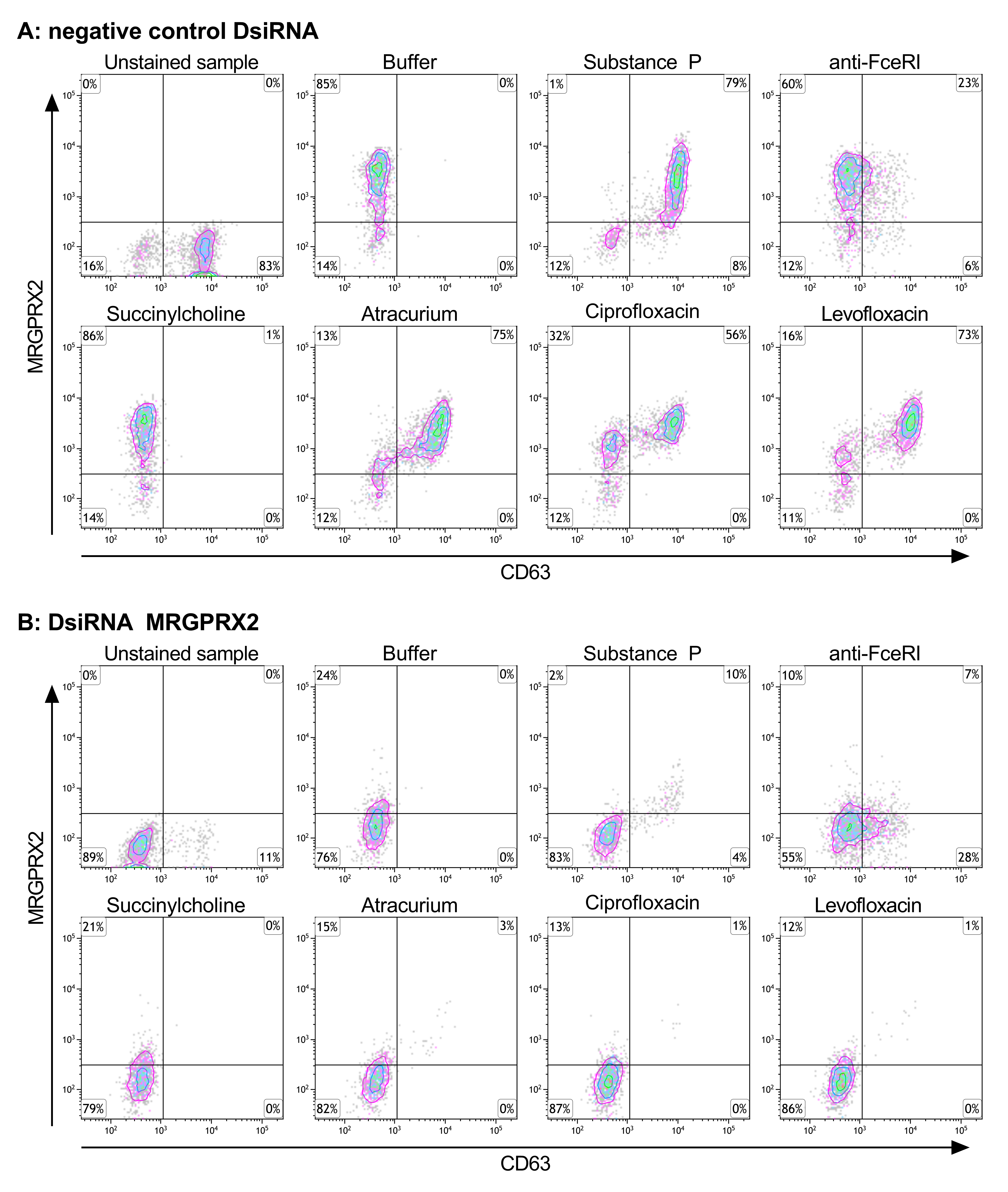

Supplement: Supplementary Figure 7 — Representative plots of CD63 up-regulation after silencing of MRGPRX2. Cells were electroporated with a negative control (A) or target specific DsiRNA (B). Thereafter, cells were incubated for 3 minutes with buffer, substance P (74 µM), anti-FcϵRI (2.5 µg/mL), succinylcholine (5536 µM), atracurium (2152 µM), ciprofloxacin (755 µM) or levofloxacin (2767 µM). MRGPRX2 silencing has no significant effect on anti-FcϵRI degranulation (17). [file Image_7.tif]

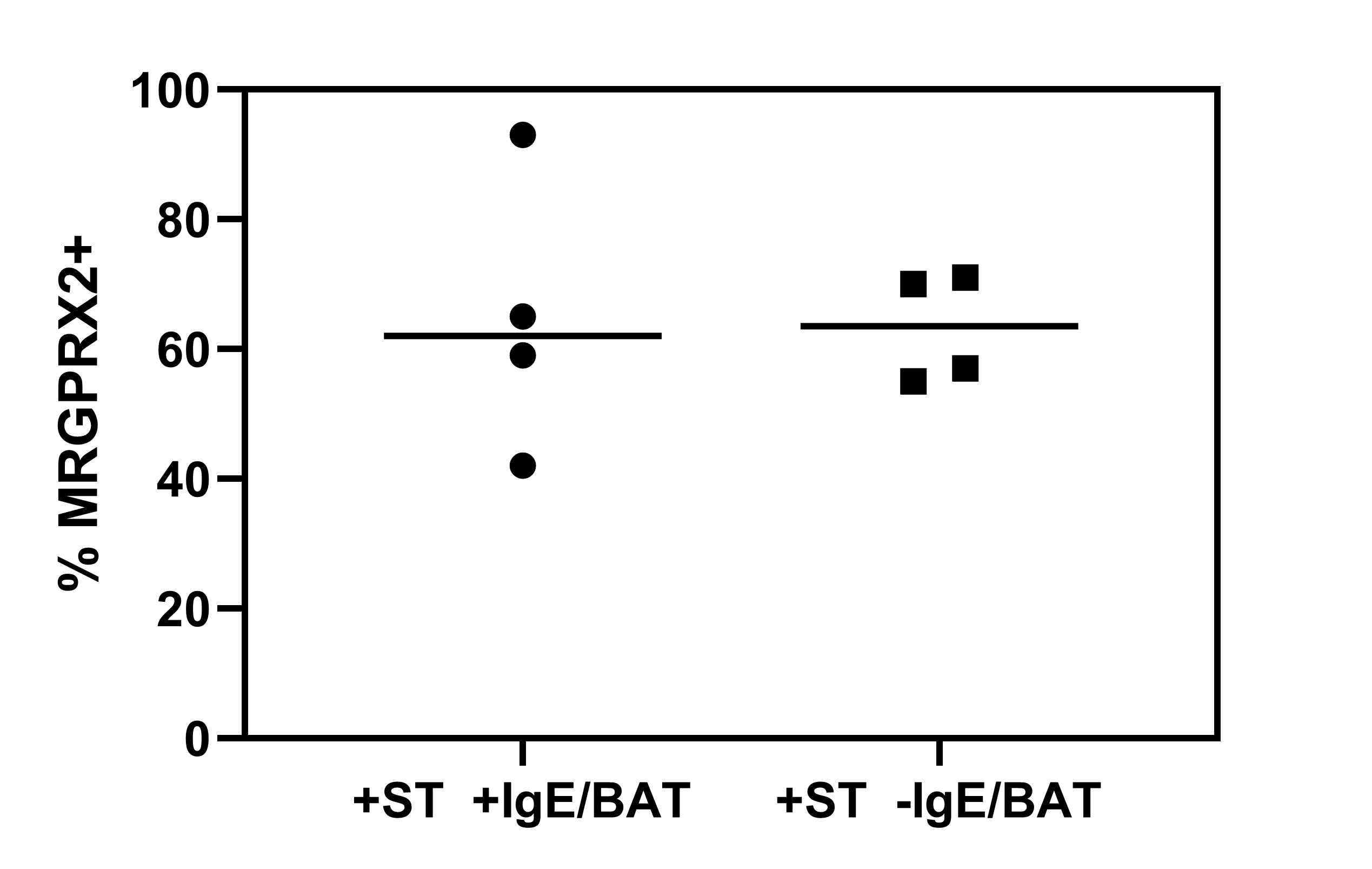

Supplement: Supplementary Figure 8 — MRGPRX2 expression on PBCMCs cultured from patients with an IgE-dependent and non IgE-mediated, i.e. probably MRGPRX2-mediated, rocuronium hypersensitivity reaction. Peripheral blood cultured mast cells (PBCMCs) are defined as CD117+CD203c+ cells. PBCMCs harbor two subpopulations: cells with surface expression of MRGPRX2 (MRGPRX2+) and cells without expression of MRGPRX2 (MRGPRX2-). [file Image_8.tif]
